# Synergistic Toxicity Reduction of Cadmium in Rice Grains by Foliar Co-Application of Nano-Silica and Surfactants

**DOI:** 10.3390/toxics13121047

**Published:** 2025-12-02

**Authors:** Jihao Kang, Pengyue Yu, Zhi Huang, Zhenglong Tong, Ruimin Chang, Zhiyan Xie, Shiyu Gui, Ying Huang

**Affiliations:** 1 Ningxiang Foliar Fertilizer Science and Technology Backyard, Hunan Hankun Industrial Co., Ltd., Changsha 420114, China; s2747502@ed.ac.uk (J.K.); yupengyue@stu.hunau.edu.cn (P.Y.); 2 National Engineering Research Center for Efficient Utilization of Soil and Fertilizer Resources, College of Resource, Hunan Agricultural University, Changsha 410128, China; huangzhi18714838265@stu.hunau.edu.cn (Z.H.); tzl13203618886@163.com (Z.T.); chang_ruimin@stu.hunau.edu.cn (R.C.); xiezhiyan@stu.hunau.edu.cn (Z.X.); guishiyu123@126.com (S.G.)

**Keywords:** rice, foliar control agent, surfactants, cadmium, heavy metal

## Abstract

Cadmium (Cd) accumulation in rice poses a serious threat to global food safety and human health. Foliar application of nano-silica (Si) offers a promising remediation strategy, but its efficacy is often limited by poor droplet retention on hydrophobic leaf surfaces. This study hypothesized that surfactants could overcome this barrier by enhancing the foliar performance of nano-Si. Through field experiments, we evaluated the synergistic effects of five surfactants (Polyvinylpyrrolidone (PVP) powder, Aerosol OT (AOT), Rhamnolipid (RH), Didecyldimethylammonium bromide (DDAB), and Alkyl Polyglycoside (APG)) when combined with nano-silica. The results demonstrated that all surfactants significantly improved wetting and retention, with alkyl polyglycoside (APG) and polyvinylpyrrolidone (PVP) being the most effective. These improvements translated into a remarkable suppression of Cd translocation within rice plants. The PVP–nano-Si combination emerged as the most potent treatment, reducing grain Cd content by 50% and achieving the lowest levels of As and Cr among all treatments. Furthermore, this synergistic effect was linked to a significant increase in grain concentrations of manganese (Mn) and zinc (Zn), which exhibit a competitive relationship with Cd. The findings reveal that surfactant co-application not only optimizes the physical application of nano-Si but also triggers beneficial nutrient–Cd interactions, providing a novel and efficient strategy for mitigating Cd contamination in rice. This study provides critical theoretical support for developing efficient and environmentally friendly foliar barrier technologies and supports safe production of rice in lightly to moderately contaminated paddy fields.

## 1. Introduction

Rice is a staple food for nearly half of the world’s population, making its safe production vital for global food security and human health. However, with the speeding up of industrialization and agricultural intensification, industrial activities, mining, and agricultural inputs have resulted in the introduction of heavy metals into farmland ecosystems. Heavy metal contamination of farmland has become a global environmental problem that threatens rice production [1]. According to a 2023 report by the Food and Agriculture Organization of the United Nations (FAO), the area of arable land contaminated by heavy metals has surpassed 40 million hectares globally, of which Asia accounts for up to 62% [2]. Particularly, the combined geological background and anthropogenic activities severely affect the rice regions in southern China [3]. Toxic heavy metals such as cadmium (Cd), arsenic (As), and lead (Pb) can enter agricultural systems through multiple pathways, including solid waste, wastewater, and atmospheric emissions. These elements are highly toxic, persistent, and prone to bioaccumulation, posing long-term risks to ecosystems [4]. Long-term intake of rice contaminated with heavy metals can lead to a series of health problems, such as food poisoning, cancer and malformation, kidney damage, etc. [5]. Therefore, it is essential to reduce the accumulation of heavy metals in rice grains to safeguard food security and human health.

Current technical means to reduce heavy metal accumulation in rice mainly include agronomic control measures, soil passivator application, foliar control agents, and planting structure adjustment [6]. Among them, foliar control agents can both improve plant nutritional status and promote crop growth in addition to significantly reducing heavy metal levels in plants [7,8,9]. Among these, foliar application of nano-silica significantly reduces cadmium content in plants [10,11,12]. For instance, foliar spraying with 0.2% SiO_2_ has been shown to lower the amount of Cd in rice from 0.35 mg/kg to 0.15 mg/kg [13]. Similarly, foliar spraying of Na_2_SiO_3_ reduced cadmium accumulation in rice by 27.1% [14]. A 2025 study revealed that the sole application of nano-silica significantly reduced Cd content in wheat roots and leaves, with root Cd levels decreasing by 16% and leaf Cd levels by 35% [15]. Nano-silicon can also help plants grow better when heavy metal pollution slows down photosynthesis. For example, application of nano-silica increases chlorophyll content and photosynthetic rates in bitter melon plants [16]. In addition, nano-silica can also reduce cadmium levels in corn [17], wheat [18], and common legume seeds [19]. The mechanism by which nano-silicon reduces Cd and other heavy metals includes silicon-enhanced cell wall lignification and co-precipitation of Cd with silicon in apoplastic spaces, thereby immobilizing Cd in vegetative tissues and restricting its remobilization to grains [10,20,21,22]. A recent study further confirmed that nano-silica application increased the proportion of Cd bound to the cell wall while decreasing its concentration in sensitive organelles, thereby reducing its toxicity and mobility within the plant [15]. Practical limitations often compromise the efficacy of conventional nano-silica foliar applications, despite their potential. The hydrophobic nature of the rice leaf surface results in poor wettability and high contact angles of aqueous droplets. This results in droplet bouncing, splashing, and rapid roll-off, culminating in low foliar deposition, short retention time, and, ultimately, insufficient and inconsistent performance of nano-silica.

Surfactants can help circumvent these barriers between surfaces. Recently, studies have demonstrated the significant role of surfactants in enhancing the foliar uptake of various substances. As amphiphilic compounds, surfactants function by reducing the surface tension of spray solutions, thereby improving wettability, penetration, and overall foliar absorption efficiency [23]. For instance, the addition of the surfactant polyvinylpyrrolidone (PVP) promoted the uptake of gold nanoparticles (AuNPs) into the parenchyma tissue of wheat leaves [24]. Similarly, research observed that surfactants markedly reduced the contact angle of droplets on rice leaves, indicating enhanced spreading and adhesion [25]. Certain surfactants, such as Silwet L-77, can further facilitate uptake by solubilizing cuticular waxes and enhancing cuticle hydration, thereby improving nanoparticle penetration into the mesophyll layer. Given these mechanisms, surfactants have been widely incorporated as key components in composite foliar barrier formulations aimed at reducing heavy metal uptake and translocation in rice. The type and concentration of the surfactant strongly influences the efficacy of such barrier systems.

Common surfactants encompass cationic, anionic, amphoteric, and nonionic surfactants [26]. Cationic quaternary ammonium salts exhibit potent antibacterial activity and gene delivery potential but raise cytotoxic and environmental concerns [27]. Anionic sulfates/sulfonates are the most produced class; they effectively reduce interfacial tension in sandstone reservoirs and act as emulsifiers in enhanced oil recovery, yet they adsorb strongly on carbonates [28]. Amphoteric betaines change charge with pH, which makes them more tolerant of salt, less irritating, and better able to work in harsh reservoir conditions [29]. Therefore, the addition of surfactants may contribute to better crop uptake of foliar control agents, thereby reducing heavy metal accumulation in plants.

Based on the above research background and current situation, the present study aims to comprehensively evaluate the efficacy of five types of surfactants: Polyvinylpyrrolidone (PVP) dust, Aerosol OT (AOT), Rhamnolipid (RH), Didecyldimethylammonium bromide (DDAB), and Alkyl Polyglycoside (APG) in enhancing the foliar application performance of nano-silica for reducing heavy metal accumulation in rice. Through field trials and simulation experiments, the specific objectives of this study are as follows: (1) To compare the effects of different surfactant types combined with nano-silica on rice foliar wettability (contact angle, rolling angle); (2) To evaluate their efficacy in reducing heavy metal accumulation in grains; (3) To reveal the mechanisms by which different surfactants regulate the foliar application behavior of nano-silicon and inhibit heavy metal accumulation in rice.

## 2. Materials and Methods

### 2.1. Experimental Materials and Design

This study utilized nano-silica (≥99.9% metals basis, 60–100 nm, nano-silicon purchased from Shanghai Aladdin Biochemical Technology Co., Ltd., Shanghai, China) along with five distinct surfactants: PVP, AOT, RH, DDAB, and APG. All surfactants were purchased from Shanghai Aladdin Biochemical Technology Co., Shanghai, China. In total, twelve treatments were established, including a control group (CK), as described in Table 1.

### 2.2. Preparation of Surfactant

A certain amount of each surfactant was weighed according to the same molar mass and dissolved in 500 mL of deionized water with magnetic stirring for 15 min until completely dispersed. Nano-silica powder (0.15 g/L) was added to the surfactant solution, followed by magnetic stirring for 15 min to obtain a stable suspension. The final volume was adjusted to 1 L using deionized water.

### 2.3. Study Area Description

The experiment was located in Huanggu Village, Liling City, Hunan Province (113.21408 E, 27.58132 N), which has a subtropical monsoon climate with high temperatures and sunshine in the summer. The soil pH is 5.04, and the contents of N, P, and K were 18.20 ± 0.22 g/kg, 0.31 ± 0.02 g/kg, and 9.40 ± 0.11 g/kg, respectively. The concentrations of toxics metals in the soil of the study area were measured as follows: As, 20.59 ± 0.59 mg/kg; Cd, 0.54 ± 0.10 mg/kg; Cr, 39.47 ± 10.47 mg/kg; Pb, 45.45 ± 4.24 mg/kg; Mn, 169.04 ± 5.97 mg/kg; and Zn, 129.7 ± 6.88 mg/kg. The experiment was arranged in a randomized complete block design with three replicates, totaling 36 plots. These plots were organized into six rows, with six plots per row. Each plot covered an area of approximately 2 m^2^. And each row was separated by a protective row to minimize cross-contamination between treatments. This experiment used the early-maturing rice variety ZhongZao 35, a widely cultivated cultivar in southern China. Each plot received a single treatment. Spray treatments were applied at the heading and maturity stages at a rate of 1 L per plot.

### 2.4. Sample Collection and Analysis

Rice plants were harvested at maturity, the attached soil was cleaned with water, and later immersed in EDTA-2Na for 10 min. The samples were then rinsed with ultrapure water and air-dried. Prior to analysis, the rice plants were dissected into roots, stems, leaves, shafts, husks, and grains. All components were then dried in an oven at 50 °C and subsequently ground into a fine powder. Plant samples (weighing 0.200 g) were digested with 8 mL of concentrated nitric acid and 2 mL of perchloric acid. The concentrations of heavy metals in the digested samples were determined using inductively coupled plasma mass spectrometry (ICP-MS, Model NEXION 350 X, Thermo Fisher Scientific, USA). To guarantee the reliability and quality of the data, standard reference sample for rice (GBW(E) 100360) from the Center of National Reference Materials of China was analyzed in addition to the plant and soil samples. The recovery of the spiked standards for each element ranged from 90 to 110% with a detection limit of 0.01 µg/mL.

Soil samples were air-dried at room temperature, crushed, and passed through a 0.145 mm sieve. Subsequently, 0.200 g of soil was digested with a mixture of HNO_3_-H_2_O_2_-HF (6:3:3) in a microwave digestion system (Model Multiwave 7501; Anton Paar (Shanghai) Trading Co., Ltd., Shanghai, China). The concentrations of heavy metals in the digested solutions were determined using inductively coupled plasma mass spectrometry (ICP-MS, Model NEXION 350X, Thermo Fisher Scientific, USA). To ensure data quality and reliability, standard reference materials for soil (GBW 07387) obtained from the Center of National Reference Materials of China were processed and analyzed alongside the samples. The recovery of the spiked standards for each element ranged from 90% to 110%, with a method detection limit of 0.01 µg/mL.

### 2.5. Analysis of Contact Angles and Rolling Angles

Contact angles and rolling angles were measured using a contact angle goniometer. The rice leaves were placed horizontally on the testing platform, and a stable suspension was deposited onto the leaf surface using a dropper pipette. The dynamic wetting process was captured using a contact angle analyzer (LSA100, LAUDA Scientific, Germany), with five consecutive images recorded over 5 s. The contact angle was determined by analyzing the final 1 s interval of the droplet–leaf interface. To measure the rolling angle, the suspension droplet was put on the flat surface of a rice leaf, and the platform was slowly tilted. The droplet morphology was monitored using a high-speed camera until incipient droplet motion was observed. The critical tilt angle at which the droplet began to roll was recorded as the roll-off angle, measured using a custom-built tilting stage (Compass Laboratory). Since the CK (deionized water) and Si (nano-silicon) treatments both slid immediately after dropping, they could not to test the rolling angle, so only the contact angle was tested.

### 2.6. Calculation of Translocation Factor

The transfer factor (*TF_A-B_*) is defined as a measure of the plant’s capacity to transport and enrich heavy metals, characterizing the migration of a substance between different tissues [30]. The calculation formula is:

(1) $${TF\;}_{A-B}\;=\;\frac{C_{B}}{C_{A}}$$

where *C_B_* (mg/kg) represents the concentration of heavy metals in specific plant tissue (stem, leaf, shaft, husk, rice) and *C_A_* (mg/kg) represents the concentration of heavy metals in the lower part of the rice system compared to *B* (root, stem, leaf, shaft, husk).

### 2.7. Data Analysis

All results were analyzed by one-way ANOVA using the SPSS 27 statistical software package. All graphics were drawn using the PC-based Origin 2024 program. Post hoc comparisons were conducted using the Duncan test in SPSS with a probability of 5% to examine the differences in treatment.

## 3. Results

### 3.1. Effect of Surfactants on Wettability of Nano-Silica

**Figure 1:**
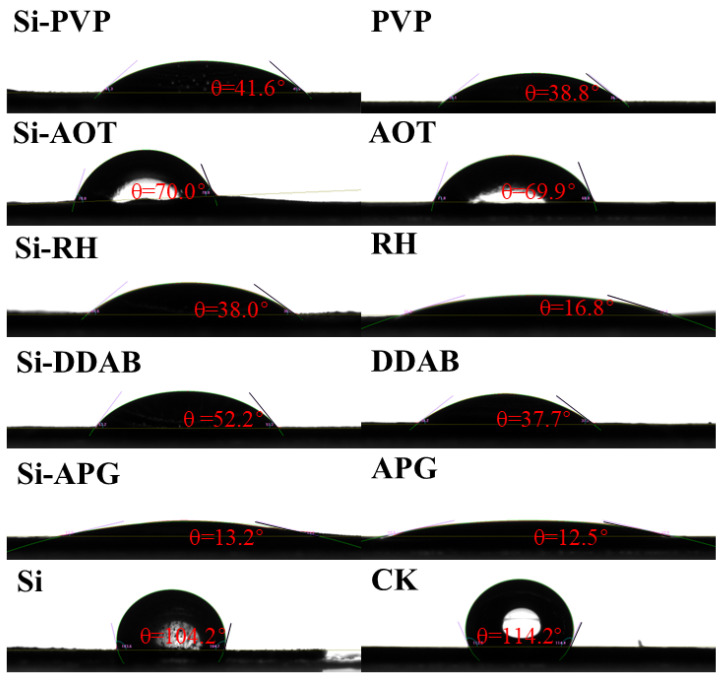
Influence of different surfactants on the contact Angle of nano-silicon. Red text indicates contact angles.

All surfactant treatments significantly improved foliar wettability compared to the CK and nano-silica alone, as shown in Figure 1. In the most effective treatments, the contact angles decreased by more than 100°. Among the surfactants applied individually, all five produced contact angles substantially lower than the CK and Si treatments, ranging from 12.5° to 69.9°. APG yielded the smallest contact angle (12.5°), indicating superior wettability, followed by RH (16.8°), while AOT yielded the largest contact angle (69.9°) within this group. It is worth noting that the synergistic effect of combining surfactants with nano-silica was remarkable. Each composite treatment significantly reduced the contact angle compared to the Si-only application. The most pronounced improvement was observed with Si-APG, which achieved a contact angle of merely 13.2°, representing a reduction of approximately 87% relative to the Si treatment. Similarly, the Si-RH and Si-PVP composites demonstrated exceptional wetting performance, reducing the contact angles to 38.0° and 41.6°, respectively. The effectiveness of the composite systems, in ascending order of the final contact angle, was Si-APG (13.2°) < Si-RH (38.0°) < Si-PVP (41.6°) < Si-DDAB (52.2°) < Si-AOT (70.0°).

### 3.2. Surfactants Exhibit a Synergistic Effect in Increasing the Rolling Angle

**Figure 2:**
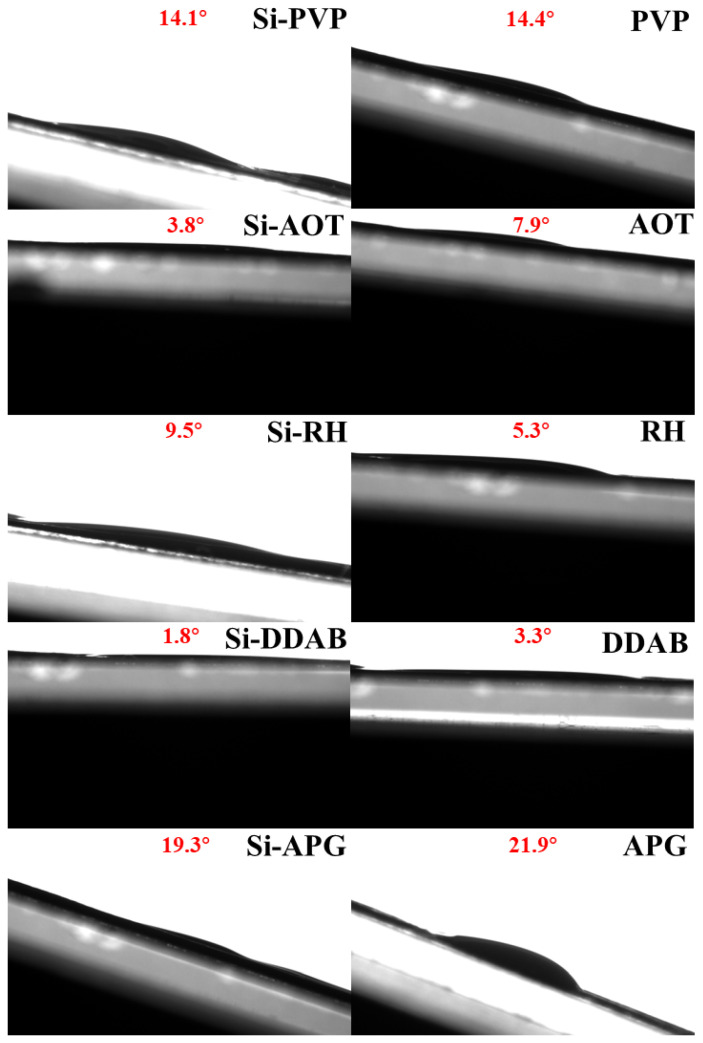
Effect of different surfactants on rolling angle of nano-silicon. Red letters indicate the rolling angle.

The rolling angle represents the dynamic retention capacity of the solution, which can be applied to the evaluation of rainwater resistance. Since CK and nano-silica alone immediately roll off upon minimal tilting of the platform, reliable measurement of their rolling angles is impossible. Therefore, the analysis focused solely on treatments containing surfactants. This study found that, except for the Si-RH composite, the rolling angles of all other surfactants combined with nano-silica were lower than those of the surfactants applied alone (Figure 2). Specifically, when surfactants PVP, AOT, RH, DDAB, and APG were applied individually, their contact angles were 14.4°, 7.9°, 5.3°, 3.3°, and 21.9°, respectively. The application of APG yielded the largest contact angle and the best adsorption effect. When surfactants were applied in combination with nano-silica, except for the RH-nano-silica composite, the addition of nano-silica reduced the rolling angles of PVP, AOT, DDAB, and APG by 2%, 51.9%, 45.5%, and 11.9%, respectively. AOT combined with nano-silica showed the largest roll-off angle reduction compared to AOT alone. The Si-APG composite system demonstrated the optimal roll-off resistance, where the effect of PVP followed closely.

### 3.3. Effect of Different Treatments in Blocking Heavy Metals in Rice

**Figure 3:**
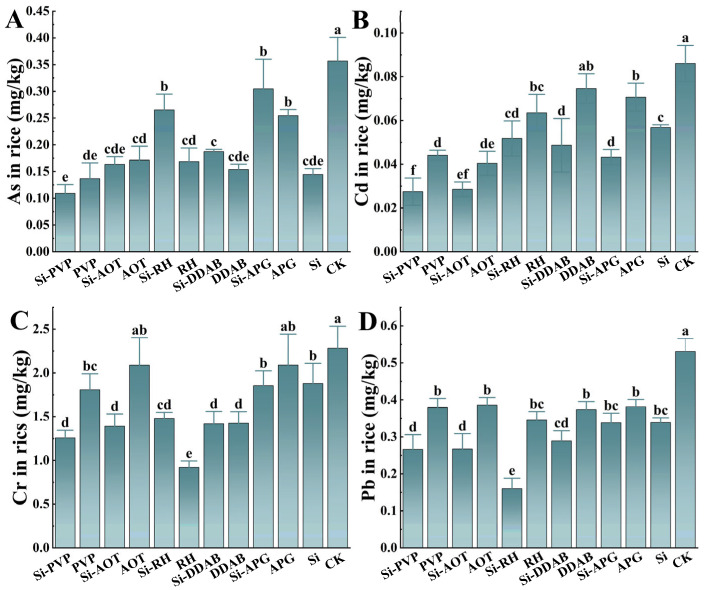
Contents of Pb, Cd, Cr and As in rice grains after spraying with different surfactants. (**A**–**D**) represent the elements As, Cd, Cr, and Pb, respectively. Different lowercase letters above the bars indicate significant differences among treatments according to Duncan’s multiple range test (*p* < 0.05).

The results showed that using only nano-silica cut the levels of Pb, Cd, Cr, and As in rice grains by 18% to 60% (Figure 3). The As, Cd, Cr, and Pb concentrations in CK grains all exceeded the national food safety limit (0.20 mg/kg for Cd and Pb, 0.35 mg/kg for As, 1.0 mg/kg for Cr, GB 2762-2022) [31], confirming the presence of heavy metal contamination in the experimental area. Among treatments with surfactants alone, AOT showed a particularly strong selective inhibition of Cd, achieving the lowest grain Cd content (0.16 mg/kg) in this group. The results indicate that combining surfactants with nano-silica further enhances the efficacy of reducing heavy metals, demonstrating a clear synergistic effect. The Si-PVP composite treatment was the most effective overall, resulting in the lowest grain contents of Cd (0.11 mg/kg), As (0.11 mg/kg), and Cr (1.26 mg/kg) among all composite treatments. In contrast, the Si-RH treatment led to the lowest Pb content (0.16 mg/kg). The Si-AOT treatment led to the highest Pb content at 0.39 mg/kg and the lowest Cd content at 0.16 mg/kg within the composite group, indicating a distinct pattern of metal-specific inhibition that varies with surfactant type.

### 3.4. Regulation of Heavy Metal Translocations Between Rice Organs by Different Treatments

**Figure 4:**
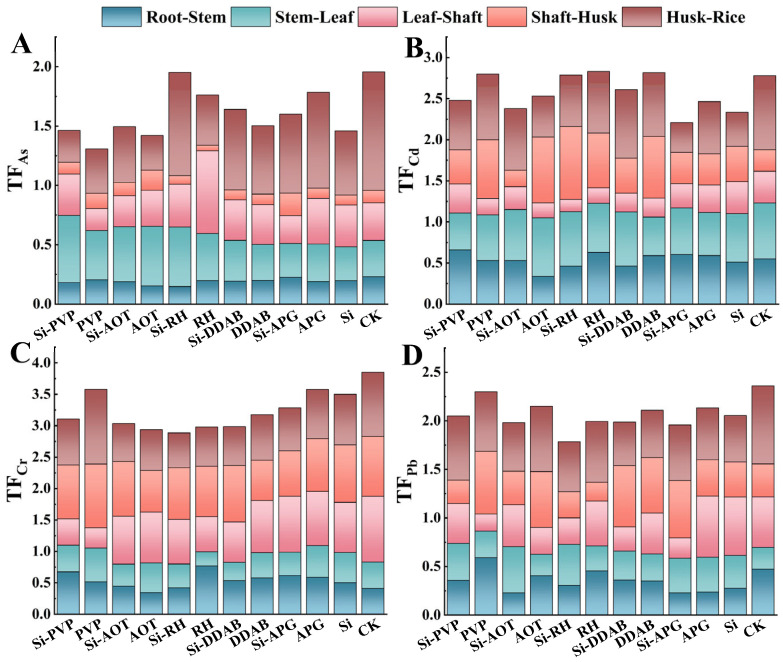
Transport capacity of Pb, Cd, Cr and As in different parts of rice after spraying with different surface activities. (**A**–**D**) represent the elements As, Cd, Cr, and Pb, respectively.

This study calculated the translocation factors (TFs) of four toxics metal elements, As, Cd, Cr, and Pb, between various parts of rice under different surfactants with nano-silica treatment. As shown in Figure 4, whether surfactants were applied alone or in combination with nano-silica, the transport coefficients of rice aboveground parts were significantly reduced, particularly in the leaf-shaft transport process. For example, Cd transport decreased by approximately 41–75%. Specifically, under PVP treatment, the transport coefficients of As, Cd, Cr, and Pb from leaves to shafts decreased to 0.18, 0.20, 0.32, and 0.17, respectively, indicating that PVP application resulted in greater heavy metal retention within leaves. Under Si-PVP treatment, the transport coefficients of As, Cd, Cr, and Pb from husk to grain were primarily reduced to 0.27, 0.6, 0.73, and 0.66, respectively. Under Si-RH treatment, the transport coefficient of arsenic from rice husks to grains increased significantly by 64% compared to the sole application of nano-silica. Under AOT treatment, the transport coefficient of As from husks to grains decreased to 0.29. Simultaneously, the transport coefficient of Cd from shafts to husks significantly increased to 0.80 compared to the control group, while the transport coefficient from husks to grains decreased to 0.50. The bio-concentration factors (BCF) from soil to roots were calculated to assess the impact on initial metal uptake (Table S1). While most treatments did not significantly alter the BCF for As, Cr, Pb, and Zn, certain treatments like Si-PVP reduced the root uptake of Cd. Nonetheless, the suppression of internal translocation remains the predominant mechanism for reducing grain metal content, as detailed in Figure 4.

### 3.5. Promotional Effects of Different Treatments on the Accumulation of (Mn, Zn)

**Figure 5:**
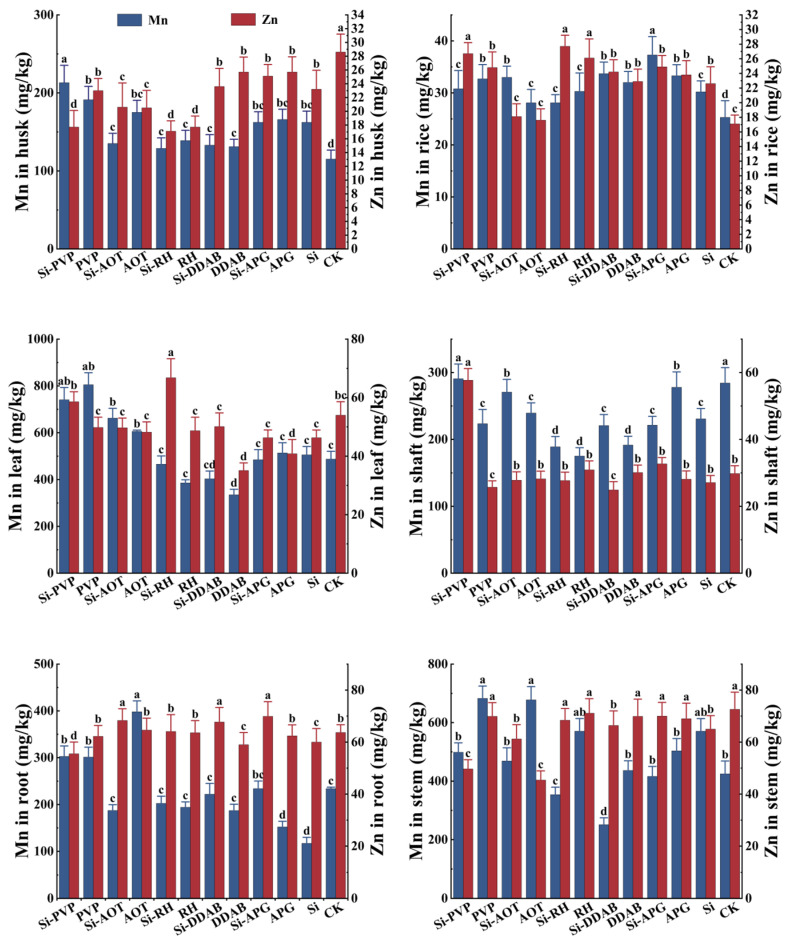
The Mn and Zn content in various parts of rice. Different lowercase letters above the bars indicate significant differences among treatments according to Duncan’s multiple range test (*p* < 0.05).

The study also determined the contents of the nutrient elements Mn and Zn in each part of the rice (Figure 5). Overall, the changes in Zn and Mn content in the underground parts of rice plants under different treatments were not significant, whereas significant changes were observed in the aboveground parts. Specifically, the application of nano-silica alone significantly increased Zn and Mn accumulation in grains compared to the CK, reaching 30.2 mg/kg and 22.6 mg/kg, respectively. Among the individual surfactant treatment groups, the treatments with the highest and lowest Mn accumulation in grains were APG (33.3 mg/kg) and AOT (17.6 mg/kg), respectively. Among the composite treatments of surfactants and nano-silica, Si-RH (27.7 mg/kg) and Si-APG (37.3 mg/kg) showed the most effective enhancement of Zn and Mn accumulation in grains, respectively. Additionally, under Si-RH treatment, leaf Zn content significantly increased to 66.8 mg/kg compared to silicon alone, while grain Zn content also rose significantly to 27.7 mg/kg. Under Si-PVP treatment, the highest Zn and Mn contents are in the shaft, reaching 57.7 mg/kg and 290.7 mg/kg, respectively.

### 3.6. Correlation Analysis Between Elements in Various Parts of Rice

**Figure 6:**
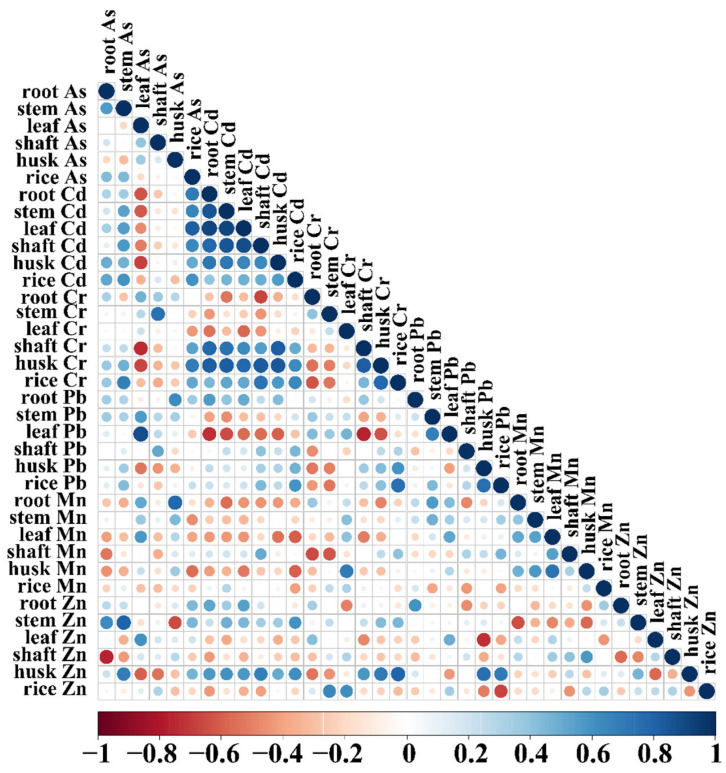
Correlation analysis between elements in various parts of rice. The heatmap displays Pearson correlation coefficients with statistical significance (*p* < 0.05), where red indicates positive correlations and blue indicates negative correlations.

To elucidate the potential mechanisms behind the reduced heavy metal content in grains, a correlation analysis was performed on the concentrations of As, Cd, Cr, Pb, Mn, and Zn across different rice tissues. As shown in Figure 6, beneficial nutrients (Mn, Zn) exhibit a significant antagonistic relationship with the toxic metal cadmium. A significant negative correlation was observed between the accumulation of Cd and that of both Mn and Zn in rice grains. The Cd content in grains exhibited the most pronounced negative correlation with the Mn content in leaves and husks. In contrast, Cd accumulation in grains showed positive correlations with the concentrations of As, Cr, and Pb, implying a synergistic co-transport or shared uptake pathway for these toxic metals. Within plant tissues, Cd accumulation in leaves was negatively correlated with the concentrations of Mn and Zn in leaves. A negative correlation was also found between Cd and Pb accumulation in the stem. The correlation between Mn and Zn accumulation in roots was not significant.

## 4. Discussion

This study demonstrates that the co-application of specific surfactants with nano-silica forms a highly effective synergistic strategy for mitigating cd accumulation in rice grains. The findings demonstrate that surfactants are not merely auxiliary additives for improving the physical properties of nano-silica but are crucial components that unlock the full potential of nano-silica as a foliar barrier. The primary synergy operates on two interconnected fronts: enhancing the foliar deposition, adhesion, and retention of the nano-silica barrier, and physiologically modulating metal transport within the plant.

### 4.1. Surfactants Enhanced the Duration and Efficacy of Nano-Silica

This study demonstrates that surfactants significantly reduce the contact angle of nano-silica solutions on the surface of rice leaves, directly enhancing droplet wettability. The hydrophobic groups of the surfactant adsorbed onto the surface of the rice leaves, while the hydrophilic groups interacted with water molecules to effectively reduce the surface tension at the liquid–solid interface, thereby promoting droplet spreading. Notably, Si-APG and APG alone achieved the smallest contact angles (12.2° and 12.5°, respectively), representing an 85.5% reduction compared to nano-silica or deionized water. This phenomenon may be attributed to the synergistic effect of the high surface activity of APG lauryl ether nanoemulsions and silica nanoparticles [32]. Alkyl polyglucosides, as bio-based nonionic surfactants, have hydrophilic groups of polyhydroxyl groups in the molecular structure that can form a hydrogen-bonding network with water and significantly enhance the solution wetting ability. On the other hand, the presence of silica nanoparticles may enhance the spreading behavior of droplets through physical adsorption or interfacial interactions [33]. Interestingly, it was also found that the contact angle of the sprayed surfactant alone (12.5°) was slightly smaller than that of its composite system with silica nanoparticles (13.2°), but the difference between the two was not significant. The result suggests that the nano-silica particles may slightly inhibit the wetting effect of surfactants, which may be due to the fact that the nano-silica particles may compete with surfactant molecules to occupy the interfacial position when adsorbed on the droplet surface, resulting in some surfactants not being able to fully play the role of lowering the surface tension. Moreover, the silica nanoparticles may increase the microscopic roughness of the droplets in contact with the leaf surface, thus slightly hindering complete wetting [32]. Nevertheless, the composite system still exhibited drastically lower contact angles than the control, confirming surfactants’ dominant role in tension reduction [32]. In addition, the difference in performance between different surfactants may be related to the molecular structure and critical micelle concentration (CMC) [34]. For example, anionic Si-AOT has a strong electrostatic repulsion between molecules, which makes it harder for micelles to form and lowers the efficiency of interfacial adsorption. In contrast, nonionic APG lauryl ether readily forms stable monolayers at interfaces, optimizing wettability.

The rolling angle, as an evaluation index of dynamic retention ability, reflects the anti-rolling performance of droplets on an inclined surface. It was found that the rolling angle of the nano-silica and surfactant composite system was generally higher than that of the surfactant alone. For example, the roll angle of the APG lauryl ether composite system increased from 19.3° (APG) to 21.9° (Si-APG), an increase of 11.9%. The observed increase in droplet viscosity can be attributed to the addition of nano-silica. It is proposed that the attached nanoparticles formed microscopic concave-convex structures, thereby increasing the contact area and mechanical anchoring effect with the leaf surface. Silica nanoparticles and surfactant molecules may form a composite film structure at the liquid-solid interface, increasing the interfacial viscosity [35]. For example, the hydrophobic chains of APG lauryl ether and the hydroxyl groups on the surface of the nano-silica may combine through hydrogen bonding or van der Waals forces to form a more stable interfacial layer, which improves the roll-off resistance. In addition, the rolling angle performance of PVP (polyvinylpyrrolidone) treatment was second only to APG lauryl ether, which may be related to the entanglement of its polymer chains [36].

### 4.2. Facilitation Mechanism of Surfactants for Heavy Metal Reduction in Rice by Nano-Silica

In this experiment, nano-silica was selected as a foliar barrier and control agent. While nano-silica alone functioned as an effective foliar barrier, reducing the accumulation of Cd and As in rice grains (Figure 3), its efficacy was significantly potentiated by coupling with specific surfactants. The Si-PVP composite demonstrated the most pronounced reduction in heavy metal content, indicating a vital partnership that extends beyond mere interfacial properties. The measurement of total silicon content in the plants (Figure S1) directly confirmed that surfactants enhance silicon uptake in rice leaves. All treatments containing nano-silica showed higher silicon levels than the CK. Furthermore, the surfactant–nano-silica composites consistently resulted in greater silicon accumulation compared to the nano-silica-alone treatment. The mitigation mechanism is likely multi-faceted. Primarily, nano-silica is known to interact with heavy metals in plants through adsorption and co-precipitation mechanisms, often forming silicates (SiO_2_·nH_2_O) that enhance the mechanical strength of cell walls and immobilize metal ions, thereby reducing their translocation [37,38,39]. The addition of PVP amplifies this effect by improving the dispersion, stability, and foliar retention of nano-silica particles, ensuring a more uniform and persistent barrier film on the leaf surface [40]. Correlation analysis indicated a clear antagonistic relationship between essential (Zn, Mn) and Cd metals. The Si-PVP treatment resulted in significantly higher concentrations of Zn and Mn in the aboveground tissues compared to the control and other treatments. Since Cd^2+^ shares transport pathways with Zn^2+^ and Mn^2+^ ions (e.g., via ZIP and NRAMP transporters), the increased uptake of these beneficial elements competitively inhibits Cd absorption and translocation [41,42,43]. The Si-PVP composite appears to favorably modulate this process, potentially by altering the physicochemical environment at the leaf surface or influencing plant signaling pathways. The ensuing upregulation of Zn transporter expression further selectively enhances Zn uptake over Cd, a well-documented defense mechanism in plants [43]. In addition, Cd stress is known to induce oxidative damage by generating reactive oxygen species (ROS), disrupting cellular homeostasis and biomolecules. Previous studies have demonstrated that silica nanoparticles can alleviate cadmium-induced oxidative stress by enhancing the activity of antioxidant enzymes such as superoxide dismutase (SOD), peroxidase (POD), and catalase (CAT) within plants [44]. In conclusion, the synergy between surfactants and nano-silica is not merely physical but also physiological. The optimal surfactant, PVP, enhances the physical barrier function and concurrently promotes the uptake of antagonistic essential elements, thereby providing a dual mechanism for effectively reducing Cd and As accumulation in rice grains. This provides a novel and strategic approach for the precise control of heavy metal contamination in moderately polluted paddy fields. And elucidating the specific enhancements in the plant’s antioxidant system and the consequent impact on grain protein content will be a future focus of our ongoing investigations into this synergistic mechanism.

## 5. Conclusions

This study comprehensively elucidates the mechanisms by which five surfactants enhance the interfacial behavior and heavy metal retention of nano-silica on rice leaf surfaces. The combination of surfactants with nano-silica extends its effective duration and enhances efficiency. This physical enhancement was directly linked to a profound inhibition of cadmium translocation to grains, with the PVP–nano-silica composite achieving a remarkable 50% reduction in grain Cd content. Beyond the physical barrier effect, our findings uncover a key physiological mechanism: the treatment promotes the accumulation of beneficial nutrients Mn and Zn in grains, which competitively inhibits Cd uptake. These results underscore the dual role of surfactants not only in optimizing the physicochemical properties of nano-silica sprays but also in modulating plant physiological pathways to suppress heavy metal uptake. Our findings provide a scientific basis for developing efficient, sustainable, and practical foliar barrier technologies aimed at ensuring rice safety in contaminated agricultural systems.

## Figures and Tables

**Table 1:** Different surfactant treatments and their main components.

Treatments	Ingredients and Concentration
Si-PVP	PVP (1 mmol/L) + nano-silicon (0.5 mmol/L)
PVP	PVP (0.4 mmol/L)
Si-AOT	AOT (1 mmol/L) + nano-silicon (0.5 mmol/L)
AOT	AOT (1 mol/L)
Si-RH	rhamnolipid (1 mmol/L) + nano-silicon (0.5 mmol/L)
RH	rhamnolipid (1 mol/L)
Si-DDAB	DDAB (1 mmol/L) + nano-silicon( 0.5 mmol/L)
DDAB	DDAB (1 mol/L)
Si-APG	APG lauryl ether nano-emulsion (1 mmol/L) + nano-silicon (0.5 mmol/L)
APG	APG lauryl ether nano-emulsion (1 mmol/L)
Si	Nano-silicon (0.5 mmol/L)
CK	Deionized water

## Data Availability

The raw data supporting the conclusions of this article will be made available by the authors on request.

## Supplementary Materials

The following supporting information can be downloaded at: https://www.mdpi.com/article/10.3390/toxics13121047/s1. Figure S1: Detection of plant total silicon content under different treatments; Table S1: Uptake coefficients of various elements from soil to rice roots; Table S2: Uptake coefficients of various elements from soil to rice root; Table S3: Transport coefficients of various elements from rice root to the stem; Table S4: Transport coefficients of various elements from rice stem to the leaf; Table S5: Transport coefficients of various elements from rice leaf to the shaft; Table S6: Transport coefficients of various elements from rice shaft to the husk; Table S7: Transport coefficients of various elements from rice husk to the rice.

## Abbreviations

The following abbreviations are used in this manuscript:

Si-PVP	Nano-silicon–Polyvinyl pyrrolidone
PVP	Polyvinyl pyrrolidone
Si-AOT	Nano-silicon–Aerosol OT
AOT	Aerosol OT
Si-RH	Nano-silicon–Rhamnolipid
RH	Rhamnolipid
Si-DDAB	Nano-silicon–Didodecyldimethylammonium Bromide
DDAB	Didodecyldimethylammonium Bromide
Si-APG	Nano-silicon–Alkyl Polyglycoside
APG	Alkyl Polyglycoside
Si	Nano-silicon
