# Supplementary material for: Synergistic Toxicity Reduction of Cadmium in Rice Grains by Foliar Co-Application of Nano-Silica and Surfactants
Source: Toxics. 2025 Dec 2;13(12):1047. doi: 10.3390/toxics13121047 (PMC12737413; doi:10.3390/toxics13121047)
Supplement: Supplementary file 1 [file toxics-13-01047-s001.zip › toxics-3996222-supplementary.pdf]

Synergistic Toxicity Reduction of Cadmium in Rice Grains by Foliar Co-Application of Nano-Silica and Surfactants

Jihao Kang [1,†], Pengyue Yu [1,2,†], Zhi Huang [2], Zhenglong Tong [2], Ruimin Chang [2], Zhiyan Xie [2], Shiyu Gui [2] and Ying Huang [1,2,*]

[1] Ningxiang Foliar Fertilizer Science and Technology Backyard, Hunan Hankun Industrial Co., Ltd., Changsha 420114, China
[2] National Engineering Research Center for Efficient Utilization of Soil and Fertilizer Resources, College of Resource, Hunan Agricultural University, Changsha 410128, China
* Correspondence: huangying@hunau.edu.cn
† These authors contributed equally to this work.

1. Number of pages: 5
2. Number of table: 7
3. Number of figure: 1

Table S1. Uptake coefficients of various elements from soil to rice roots.

	As	Cd	Cr	Pb	Mn	Zn
Si-PVP	5.682856	2.12963	0.35622	0.667767	1.646845	0.427911
PVP	7.651287	2.203704	0.424373	0.743014	2.265848	0.479568
Si-AOT	6.510442	4.055556	0.399797	0.735974	2.502403	0.5266
AOT	7.173871	4.240741	0.490752	0.89637	1.703015	0.498072
Si-RH	7.862555	3.685185	0.530783	0.721452	1.748888	0.494217
RH	7.737251	3.148148	0.480872	0.835424	2.939207	0.490362
Si-DDAB	7.10442	3.759259	0.467444	0.754895	1.131532	0.521974
DDAB	7.051481	3.481481	0.405371	0.711771	2.332443	0.454896
Si-APG	7.169014	5.314815	0.390676	0.919692	1.778632	0.538936
APG	7.475474	5.203704	0.314923	0.784378	3.309007	0.48111
Si	7.514327	4.425926	0.334685	0.752695	4.875652	0.462606
CK	7.563866	4.888889	0.372435	0.780198	1.816588	0.491133

Table S2. Uptake coefficients of various elements from soil to rice root.

	As	Cd	Cr	Pb	Mn	Zn
Si-PVP	5.682856	2.12963	0.35622	0.667767	1.646845	0.427911
PVP	7.651287	2.203704	0.424373	0.743014	2.265848	0.479568
Si-AOT	6.510442	4.055556	0.399797	0.735974	2.502403	0.5266
AOT	7.173871	4.240741	0.490752	0.89637	1.703015	0.498072
Si-RH	7.862555	3.685185	0.530783	0.721452	1.748888	0.494217
RH	7.737251	3.148148	0.480872	0.835424	2.939207	0.490362
Si-DDAB	7.10442	3.759259	0.467444	0.754895	1.131532	0.521974
DDAB	7.051481	3.481481	0.405371	0.711771	2.332443	0.454896
Si-APG	7.169014	5.314815	0.390676	0.919692	1.778632	0.538936
APG	7.475474	5.203704	0.314923	0.784378	3.309007	0.48111
Si	7.514327	4.425926	0.334685	0.752695	4.875652	0.462606
CK	7.563866	4.888889	0.372435	0.780198	1.816588	0.491133

Table S3. Transport coefficients of various elements from rice root to the stem.

	As	Cd	Cr	Pb	Mn	Zn
Si-PVP	0.180583	0.66087	0.674253	0.358484	1.646845	0.895495
PVP	0.205408	0.529412	0.515821	0.591649	2.265848	1.123794
Si-AOT	0.188736	0.52968	0.444233	0.227803	2.502403	0.896047
AOT	0.152055	0.336245	0.342282	0.404762	1.703015	0.702786
Si-RH	0.147508	0.462312	0.421957	0.304971	1.748888	1.067083
RH	0.198481	0.629412	0.769758	0.453779	2.939207	1.117925
Si-DDAB	0.193465	0.463054	0.536585	0.361702	1.131532	0.980798
DDAB	0.199738	0.590426	0.576875	0.351468	2.332443	1.184746
Si-APG	0.224714	0.602787	0.615435	0.22823	1.778632	1.001431
APG	0.190294	0.590747	0.586484	0.236185	3.309007	1.105769
Si	0.197906	0.51046	0.501893	0.277112	4.875652	1.083333
CK	0.230577	0.549242	0.412245	0.473209	1.816588	1.139717

Table S4. Transport coefficients of various elements from rice stem to the leaf.

	As	Cd	Cr	Pb	Mn	Zn
Si-PVP	0.565547	0.447368	0.422996	0.380515	1.486459	1.179074
PVP	0.413473	0.555556	0.53588	0.272773	1.179435	0.712446
Si-AOT	0.463241	0.62069	0.352354	0.475066	1.413057	0.812092
AOT	0.504007	0.714286	0.472097	0.22074	0.893626	1.061674
Si-RH	0.502094	0.663043	0.377828	0.423	1.315715	0.976608
RH	0.396901	0.598131	0.224504	0.25769	0.675898	0.684951
Si-DDAB	0.343463	0.659574	0.287879	0.298147	1.604299	0.754518
DDAB	0.303103	0.468468	0.40195	0.277045	0.767186	0.502146
Si-APG	0.286102	0.566474	0.370917	0.358491	1.164344	0.661429
APG	0.314783	0.524096	0.504801	0.361045	1.020465	0.591304
Si	0.283801	0.590164	0.479638	0.335443	0.886415	0.712308
CK	0.305764	0.682759	0.417492	0.222884	1.147093	0.743802

Table S5. Transport coefficients of various elements from rice leaf to the shaft.

	As	Cd	Cr	Pb	Mn	Zn
Si-PVP	0.349791	0.352941	0.421446	0.410628	0.392308	0.984642
PVP	0.184604	0.2	0.323974	0.176147	0.277571	0.516064
Si-AOT	0.261945	0.277778	0.761134	0.433702	0.408727	0.559356
AOT	0.30212	0.181818	0.811502	0.277473	0.395245	0.585062
Si-RH	0.359466	0.147541	0.709581	0.271868	0.4058	0.414671
RH	0.696414	0.1875	0.557927	0.461712	0.454098	0.634497
Si-DDAB	0.340535	0.225806	0.642105	0.248649	0.547643	0.497006
DDAB	0.335609	0.230769	0.830189	0.422222	0.571983	0.85755
Si-APG	0.23393	0.295918	0.892045	0.207602	0.456665	0.706263
APG	0.385033	0.333333	0.861413	0.628289	0.541083	0.688725
Si	0.35328	0.388889	0.798742	0.603774	0.456199	0.585313
CK	0.317851	0.383838	1.047431	0.52139	0.583299	0.551852

Table S6. Transport coefficients of various elements from rice shaft to the husk.

	As	Cd	Cr	Pb	Mn	Zn
Si-PVP	0.098086	0.416667	0.857988	0.241176	0.733058	0.306759
PVP	0.129555	0.714286	1.013333	0.645833	0.855928	0.894942
Si-AOT	0.110749	0.2	0.87234	0.343949	0.499076	0.741007
AOT	0.169591	0.8	0.661417	0.574257	0.731412	0.72695
Si-RH	0.071926	0.888889	0.822785	0.269565	0.682372	0.617329
RH	0.045767	0.666667	0.803279	0.195122	0.793261	0.572816
Si-DDAB	0.084592	0.428571	0.901639	0.630435	0.602628	0.947791
DDAB	0.088136	0.75	0.642857	0.571429	0.684595	0.853821
Si-APG	0.189189	0.37931	0.72293	0.591549	0.733394	0.767584
APG	0.087324	0.37931	0.839117	0.371728	0.596977	0.914591
Si	0.084691	0.428571	0.913386	0.359375	0.703511	0.856089
CK	0.103152	0.263158	0.950943	0.338462	0.405206	0.959732

Table S7. Transport coefficients of various elements from rice husk to the rice.

	As	Cd	Cr	Pb	Mn	Zn
Si-PVP	0.268293	0.6	0.731034	0.658537	0.144533	1.508475
PVP	0.375	0.8	1.190789	0.612903	0.170936	1.078261
Si-AOT	0.470588	0.75	0.603659	0.5	0.244264	0.878641
AOT	0.293103	0.5	0.64881	0.672414	0.16048	0.858537
Si-RH	0.870968	0.625	0.553846	0.516129	0.217998	1.619883
RH	0.425	0.75	0.62585	0.625	0.218143	1.474576
Si-DDAB	0.678571	0.833333	0.618182	0.448276	0.253383	1.025424
DDAB	0.576923	0.777778	0.722222	0.486842	0.244088	0.891051
Si-APG	0.666667	0.363636	0.682819	0.571429	0.229821	0.992032
APG	0.806452	0.636364	0.785714	0.535211	0.200723	0.92607
Si	0.538462	0.416667	0.806034	0.478261	0.186137	0.974138
CK	1	0.9	1.02381	0.80303	0.219618	0.597902

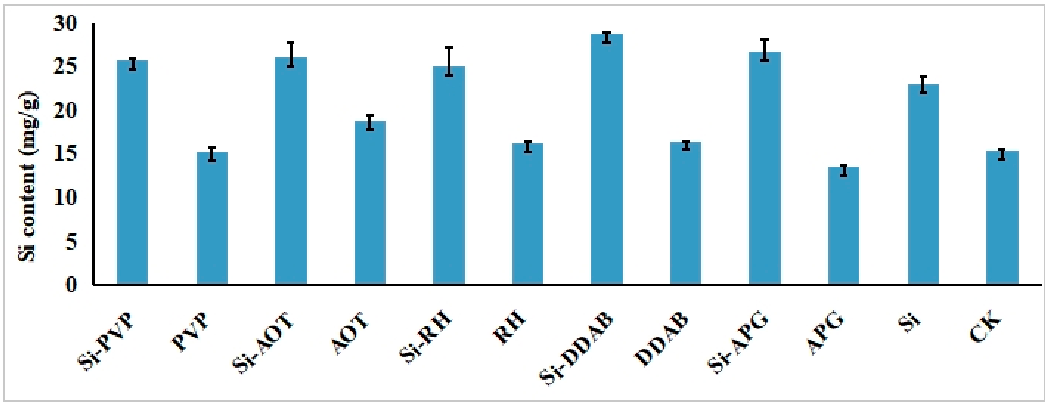

Figure S1. Detection of plant total silicon content under different treatments.
